# Prognostic role of myeloid-derived suppressor cells in cancers: a systematic review and meta-analysis

**DOI:** 10.1186/s12885-018-5086-y

**Published:** 2018-12-05

**Authors:** Lisha Ai, Shidai Mu, Yadan Wang, Huafang Wang, Li Cai, Wenzhu Li, Yu Hu

**Affiliations:** 1 Institute of Hematology, Union Hospital, Tongji Medical College, Huazhong University of Science and Technology, Wuhan, 430022 China; 2 Institute of Geriatrics, Union Hospital, Tongji Medical College, Huazhong University of Science and Technology, Wuhan, 430022 China

**Keywords:** Myeloid derived suppressor cells, Meta-analysis, Prognosis

## Abstract

**Background:**

Myeloid-derived suppressor cells (MDSCs) is a heterogeneous population of immature myeloid cells, inhibiting both the innate and adaptive immunity. Recent studies validated that MDSCs caused immune suppression and promoted cancer progression through various mechanisms. However, the prognostic value of MDSCs in cancer remains controversial.

**Methods:**

Here, we performed this meta-analysis to evaluate the prognostic value of MDSCs in various types of cancer. The electric databases, such as Pubmed, Embase and Web of Science, were searched for relevant publications. Hazards ratios (HRs) with the corresponding 95% confidence intervals (95%CIs) were calculated to evaluate the prognostic role of MDSCs in cancer.

**Results:**

A total of 16 studies with 1864 patients were enrolled in our meta-analysis. Elevated MDSCs frequency was shown to be associated with shorter overall survival (OS) (HR = 2.46, 95%CI: 1.87–3.23), and poor disease-free survival / recurrence-free survival (DFS / RFS) (HR = 3.26, 95%CI: 2.10–5.04) after treatment. Furthermore, similar results were also observed in the stratified subgroup analysis, which included the analysis by region, sample size, cancer type, NOS scores, subtype and cut-off value of MDSCs.

**Conclusion:**

High MDSCs might be related to poor clinical outcomes of patients with cancer, that is, MDSCs might be a potential prognostic biomarker in cancer.

## Background

Cancers have become the most frequent cause of death worldwide due to rapid progress, with approximately 1650 Americans per day estimated to die in 2017. Generally, therapies including chemotherapy, radiotherapy, surgical resection and immunotherapy were applied to treat different types of cancers [1], resulting in an overall drop of 25% in cancer death rates over 2 decades. However, the prognosis of most cancers remains poor. Recent findings have validated the importance of immunosuppressive network in the carcinogenesis and progression via suppressing antitumor immune system, thus leading to tumor invasion [2]. And there are rising studies on the clinical significance of immunosuppressive parameters in various cancers.

Myeloid-derived suppressor cells (MDSCs), a heterogeneous population of immature myeloid cells, are well-known to suppress both innate and adaptive immunity. The most common phenotype of MDSCs can be characterized as CD11b^+^CD33^+^HLA-DR^−/low^, which include monocytic CD14^+^CD15^−^ and granulocytic CD14^−^CD15^+^ subtypes [3]. In cancer patients, MDSC expansion inhibits T cell proliferation, decreases cytokines secretion, and recruiting regulatory T cells, etc., thus hampering the host anti-tumor immune response [3–7].

Currently, accumulating studies have investigated the role of MDSCs in both solid tumor and hematologic malignances, and MDSCs was found to be an independent prognostic factor in melanoma [8, 9], gastrointestinal (GI) cancers [10–12], NK/T lymphoma [13], bladder cancer [14] and so on. However, increasing researches referring to MDSCs and cancer questioned the reliability of MDSCs acting as a prognostic biomarker in various malignancies [15, 16]. Here, a meta-analysis was first performed to estimate the correlation between MDSCs and the survival outcomes of cancer patients, providing a basis for the predicting role of MDSCs in the various cancers.

## Methods

### Search strategy

A systematic search was conducted in 3 electric databases including PubMed (Medline), ISI Web of Science, and Ovid (EMBASE) databases. All relevant publications were picked out until January 2018. The search strategies are as following: “MDSC” (e.g. “Myeloid-derived suppressor cells”), “prognosis” (e.g. “survival” “mortality” “outcome”) and “cancer” (e.g. “tumor” “carcinoma” “neoplasm” “leukemia” “lymphoma” “myeloma”). Furthermore, the reference lists of retrieved studies were also checked to find more studies.

### Selection criteria

The inclusion criteria of this meta-analysis were: (1) Patients were histopathologically diagnosed with cancer; (2) Association between the pretreatment MDSCs level and clinicopathological parameters including OS, PFS, etc. was reported; (3) Studies were also allowed if we could reconstruct HRs and 95%CIs from the Kaplan-Meier survival curves or by other methods reported [17]. Exclusion criteria of this meta-analysis were: (1) conference abstracts, case reports, reviews, etc.; (2) publications with insufficient information for meta-analysis; (3) multiple published reports. We enrolled the most recent publication concerning the same cohort in our meta-analysis.

### Data extraction

Two reviewers (Shidai Mu and Yadan Wang) independently screened the studies for eligibility. Two reviewers (Lisha Ai and Yu Hu) evaluated the quality, and extracted the data from eligible studies. The Newcastle–Ottawa Quality Assessment Scale (NOS) was applied to assess the quality of the include studies [18]. Studies which got ≥ 7 in the NOS were assigned as high-quality. A predefined table was used to list the following relevant data: (1) characteristics of each study, such as the first author’s name, sample size, the country in which the study was carried out, year of publication, age of patients, gender, and follow-up period; (2) survival data including OS, and DFS/RFS (OS was defined as the length of time from either the date of diagnosis or the start of treatment for a disease until the death of patient or the last follow-up. DFS / RFS was defined as the length of time after primary treatment for a cancer until the patient survived without any signs or symptoms of that cancer); (3) cut-off value defining “elevated MDSCs”.

### Statistical analysis

Hazard ratio (HR) and 95% confidence intervals (95%CIs) were mostly extracted from the reports of studies, otherwise we estimates HRs and 95CIs according to the methods published by Parmer et al. [17]. The χ^2^-based Q test and I^2^ test were used to check the heterogeneity among included studies [19]. We used the fixed-effect model for analysis if no significant heterogeneity was found between studies (*p* > 0.10, I^2^ < 30%). The source of heterogeneity was further explored by subgroup analysis and galbraith plots.

A sensitivity analysis was performed to to determine which parameters have the greatest impact on the overall results. Result was regarded to be significant when *p* < 5%. The Begg’s funnel plot was used to assess publication bias. And the trim and fill analysis was performed when the possibility of publication bias was significant. All analyses were carried out using STATA statistical software 14.0 (STATA, College Station, TX).

## Results

### Selection and characteristics of the included studies

**Fig. 1:**
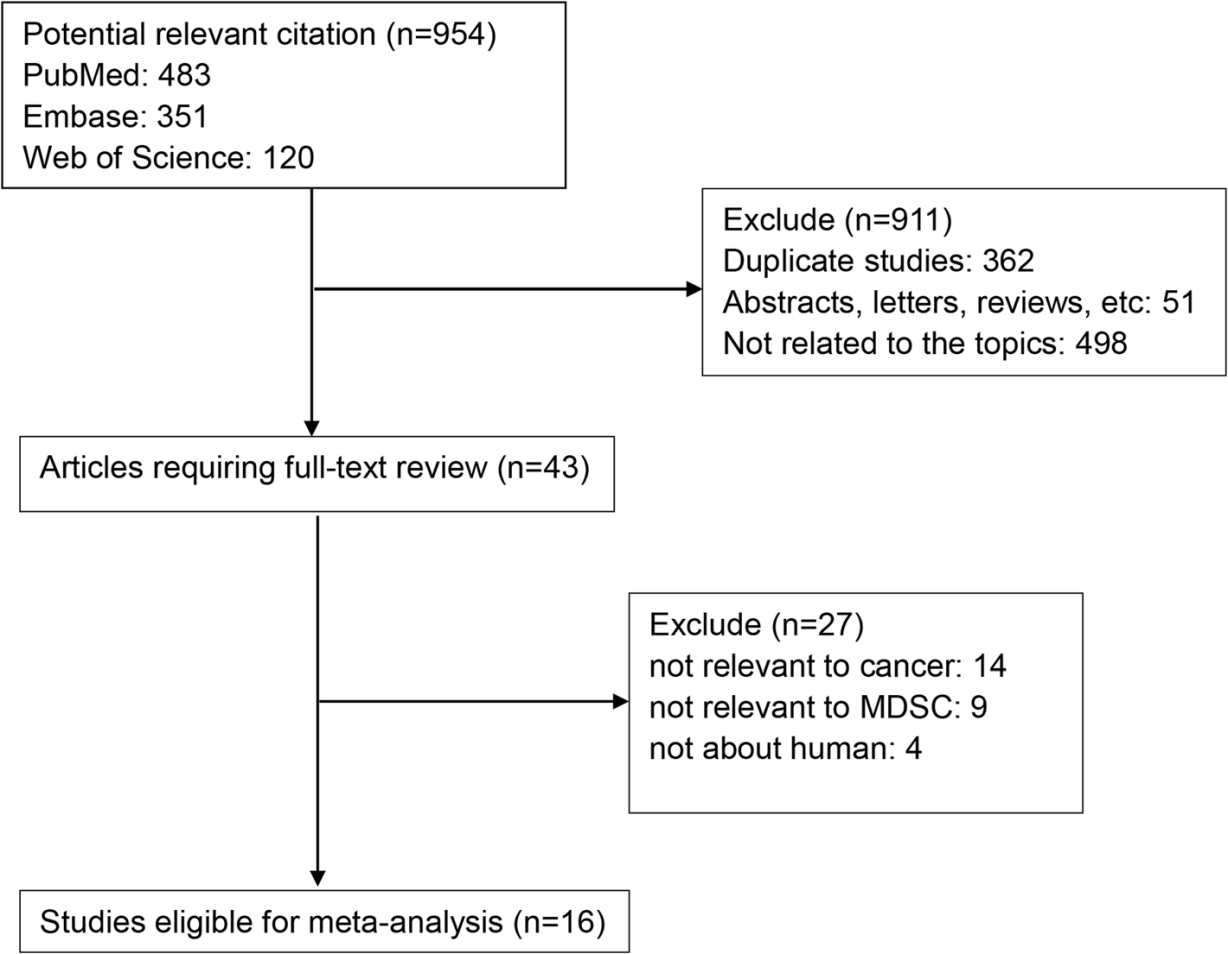
Flow diagram of selecting relevant studies included in the meta-analysis

As shown in Fig. 1, a total of 954 studies were retrieved with our search algorithm. After excluding the duplicates (*n* = 362); abstracts, case reports, reviews, etc. (*n* = 51); and other unrelated studies (*n* = 498), the rest studies (*n* = 43) were then assessed by reading the full text. Additional studies without specific data concerning cancer (*n* = 14) nor MDSCs (*n* = 9) or not about human (n = 4) were also excluded,. Therefore, 16 studies between 2011 and 2017 with a total of 1864 cancer patients were enrolled in this meta-analysis.

Table 1 showed the summary on the characteristics of the included studies. Thirteen studies were from the eastern region and six from the western region. Seven studies enrolled < 50 patients, 12 studies had > 50 patients. Nine studies were of high quality because NOS score was above 7. Nineteen studies had data for OS, 3 studies for RFS, and 3 studies for DFS. Additionally, three articles reported the different MDSCs levels in patients before and after therapy.

**Table 1:** Characteristics of studies included in the meta-analysis

Study	Year	Country	Sample size	Cancer types	Phenotype	Cut-off	Age	Follow-up (month)	NOS scores	Survival analysis
Zhang, Y	2017	China	76	rectal carcinoma	HLA-DR^−^CD33^+^CD11b^+^	3.68	NA	NA	5	OS
Yang, G	2017	China	113	bladder cancer	HLA-DR^−^CD33^low^CD11b^+^CD3^−^	21	66.5 (45–84)	43 (4–60)	6	OS
Gao, X.H	2017	China	183	HCC	HLA-DR^-/low^CD14^−^	2.31	NA	24 (1.3–28.8)	6	OS, RFS
Wang, D1	2016	China	92	HCC	HLA-DR^-/low^CD14^−^	14.6	NA	NA	6	OS
Wang, D2	2016	China	92	HCC	HLA-DR^-/low^CD14^−^	14.6	NA	NA	6	OS
Choi, H.S	2016	Korea	28	GC	HLA-DR^−^CD11b^+^CD14^+^CD45^+^	2.2	37–88	29 (10–42)	8	OS, DFS
Zhang, H1	2015	China	32	NK/T lymphoma	HLA-DR^−^CD33^+^CD11b^+^	1.1	40.5 (17–70)	52	7	OS, DFS
Zhang, H2	2015	China	32	NK/T lymphoma	HLA-DR^−^CD33^+^CD11b^+^CD14^+^	0.7	NA	NA	7	OS, DFS
Yuan, L1	2015	China	64	rectal carcinoma	Lin^−^HLA-DR^−^CD33^+^CD11b^+^	3.78	62 (38–76)	72	5	OS
Yuan, L2	2015	China	64	rectal carcinoma	Lin^−^HLA-DR^−^CD33^+^CD11b^+^	2.11	NA	NA	5	OS
Tian, T	2015	China	42	small-cell lung cancer	HLA-DR^-/low^CD14^−^	21.7	62.4	36	6	OS
Jiang, H	2015	Germany	51	advanced melanoma	HLA-DR^−^CD11b^+^CD14^+^CD15^−^	2.3	61.26 (33–88)	7	8	OS
Huang, H	2015	China	78	ESCC	HLA-DR^-/low^CD14^−^	2.38	62.4 (46–77)	42	7	OS
Chevolet, I	2015	Belgium	69	melanoma	Lin^−^HLA-DR^−^CD33^+^CD11b^+^	4.13	NA	39	7	OS
Weide, B	2014	Australia	94	advanced melanoma	HLA-DR^-/low^CD11b^+^CD14^+^	11	NA	15	9	OS
Wang, L	2013	Singapore	40	GC	Lin^−^HLA-DR^−^CD33^+^	4	NA	NA	7	OS
Arihara, F1	2013	Japan	123	HCC	HLA-DR^-/low^CD14^−^	22	NA	NA	7	RFS-U
Arihara, F2	2013	Japan	123	HCC	HLA-DR^-/low^CD14^−^	22	NA	NA	7	RFS-U, RFS-M
Solito, S1	2011	Italy	25	colorectal cancer	Lin^−^HLA-DR^−^CD33^+^CD11b^+^	2.54	NA	NA	9	OS
Solito, S2	2011	Italy	26	bresat cancer	Lin^−^HLA-DR^−^CD33^+^CD11b^+^	3.17	NA	NA	9	OS
Gabitass, R.F	2011	UK	256	pancreatic, esophageal and gastric cancer	Lin^low/-^HLA-DR^−^CD33^+^CD11b^+^	2	NA	NA	7	OS

*HCC* hepatocelluar carcinoma, *GC* gastric cancer, *ESCC* esophageal squamous cell carcinoma

*NA* not applicable, *NOS* the Newcastle–Ottawa Quality Assessment Scale

*OS* overall survival, *DFS* disease-free survival, *RFS* recurrence-free survival, *U&M* univariate & multivariate survival analysis

### Association between MDSCs and survival of cancer patients

**Fig. 2:**
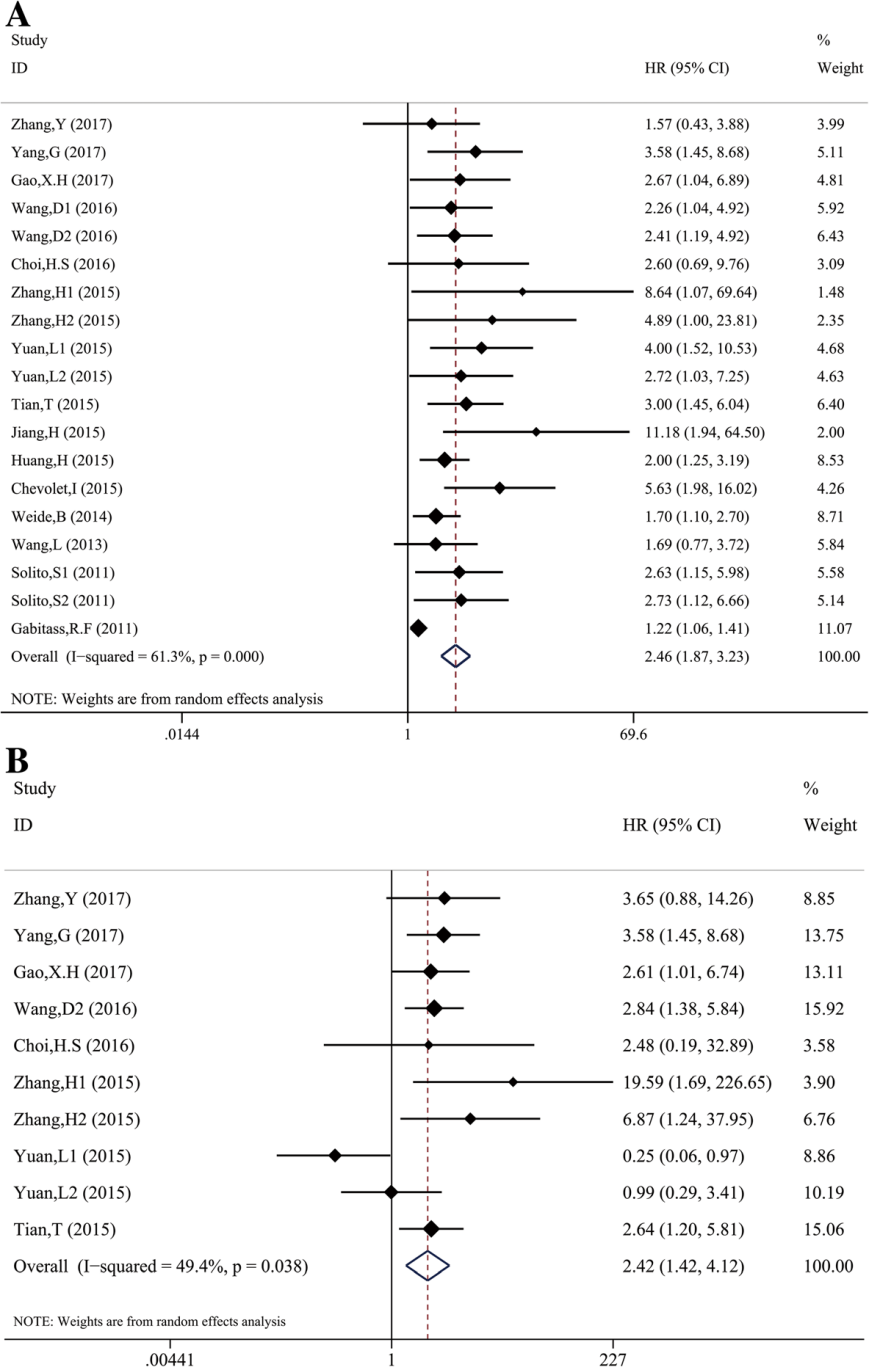
Meta-analysis of the association between elevated MDSC and OS in cancer. **a** Univariate analysis, **b** Multivariate analysis

Nineteen studies examined the correlation between MDSCs and survival of cancer patients. With significant heterogeneity (χ^2^ = 46.5, *p* < 0.01; I^2^ = 61.3%), the pooled HR 2.46 (95%CI: 1.87–3.23) indicated that cancer patients with higher MDSCs frequency might have shorter OS (Fig. 2). The combined HR was 2.42 (95%CI: 1.42, 4.12) from the results of multivariate analysis, indicating that MDSCs was an independent prognostic factor of OS in cancer patients.

Subgroup analysis was stratified by the region, sample size, cancer types, subtypes of MDSCs, cut-off value defining elevated MDSCs and NOS score, in order to explore the source of heterogeneity. As shown in Table 2, the subgroup analysis did not change the prognostic effects of MDSCs in predicting the OS of cancer patients.

**Table 2:** Subgroup analysis for overall survival in cancer patients with higher MDSCs

Subgroup analysis	No. of studies	No. of patients	Pooled HR(95%CI)		Heterogeneity	
			Fixed	Random	I^2^	*p*-value
Region						
Eastern	13	936	2.47 (1.95,3.12)	2.47 (1.95,3.12)	0%	0.892
Western	6	521	1.35 (1.18,1.54)	2.37 (1.41,3.97)	75.8%	0.001
Sample size						
<50	7	225	2.67 (1.85,3.84)	2.67 (1.85,3.84)	0%	0.786
≥50, < 100	9	680	2.26 (1.78,2.88)	2.37 (1.78,3.14)	18.9%	0.275
≥100	3	552	1.27 (1.11,1.46)	2.04 (0.95,4.37)	74.5%	0.020
Cancer types						
GI cancers	8	631	1.36 (1.20,1.55)	1.92 (1.36,2.72)	54.2%	0.033
HCC	3	367	2.41 (1.53,3.82)	2.41 (1.53,3.82)	0%	0.965
NKT	2	64	6.02 (1.70,21.28)	6.02 (1.70,21.28)	0%	0.670
Melanoma	3	214	2.24(1.50,3.35)	3.87 (1.24,12.04)	73.9%	0.022
Other types	3	181	3.07 (1.91,4.92)	3.07 (1.91,4.92)	0%	0.912
Subtypes						
Total-MDSCs	10	765	1.40 (1.23,1.60)	2.53 (1.63,3.94)	68.4%	0.001
PMN-MDSCs	5	487	2.32 (1.73,3.13)	2.32 (1.73,3.13)	0%	0.912
Mo-MDSCs	4	205	2.08 (1.40,3.11)	2.95 (1.34,6.51)	45.9%	0.136
Cut-off value						
<10	14	1024	1.46 (1.29,1.66)	2.56 (1.79,3.67)	64.3%	0.001
≥10	5	433	2.23 (1.67,2.98)	2.23 (1.67,2.98)	0%	0.527
NOS score						
<7	10	901	2.59 (2.02,3.31)	2.59 (2.02,3.31)	0%	0.770
≥7	9	556	2.17 (1.45,3.25)	1.36 (1.19,1.55)	60.5%	0.009

**Fig. 3:**
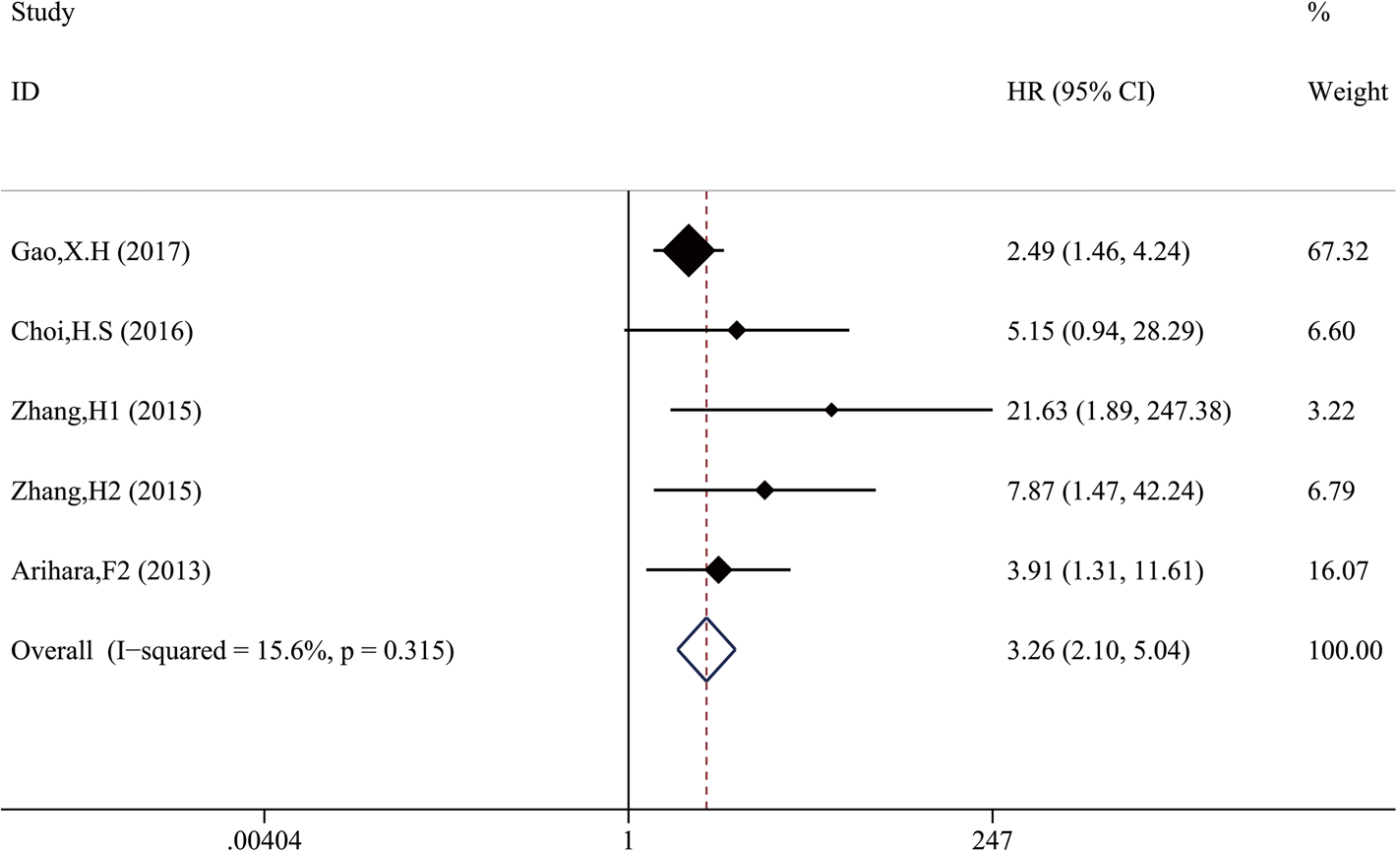
Meta-analysis of the association between elevated MDSCs and DFS/RFS in cancer

Furthermore, 306 patients from 2 studies reported RFS and 92 patients from 3 studies reported DFS. Significant correlation between high MDSCs and shorter RFS/DFS (HR 3.66, 95%CI: 2.10–6.37) was shown with low heterogeneity (χ^2^ = 4.74, *p* = 0.315; I^2^ = 15.6%) (Fig. 3). Because multivariate survival analysis was applied in these trails above, our combined HR and 95%CIs further validated the independent prognostic role of MDSCs in predicting RFS and DFS.

### Sensitivity analyses

**Fig. 4:**
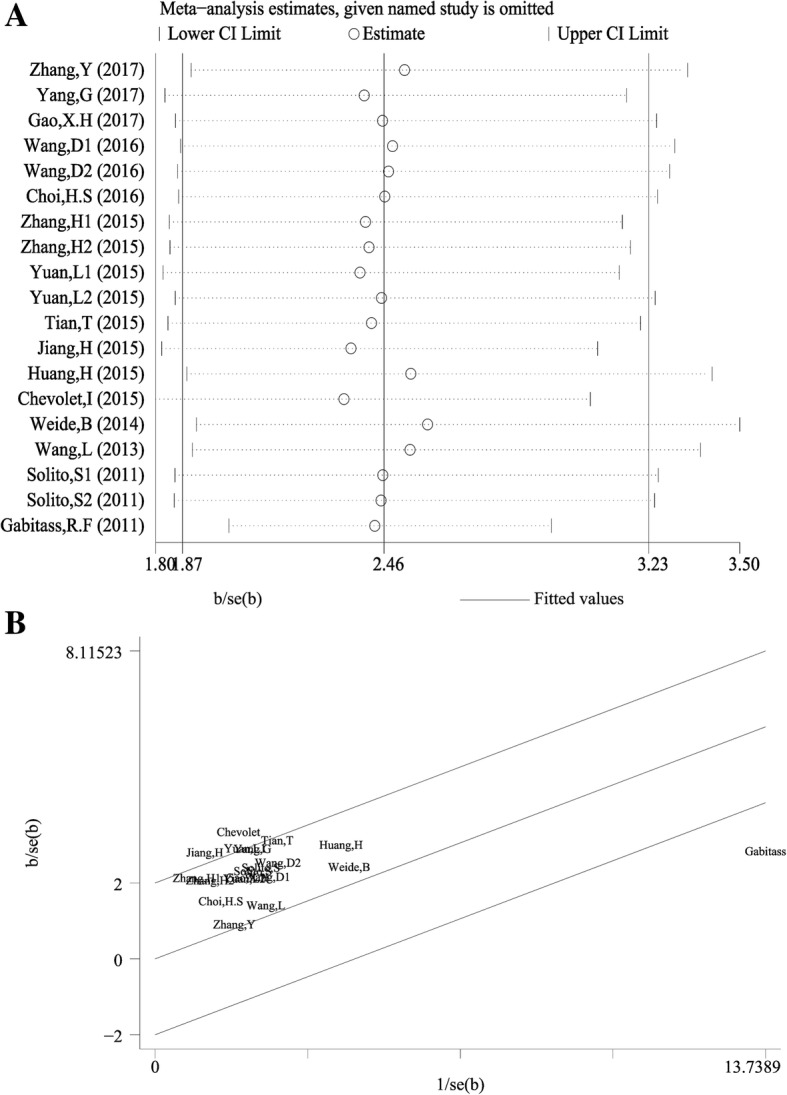
Sensitivity analysis of the enrolled studies. **a** Influence analysis, **b** Galbraith plot

As shown in Fig. 4, one study from Gabitass, R.F et al., 2011 affected the results obviously, which was possibly the main origin of heterogeneity to some extent. After delete this study, the pooled HR was 2.43 (95%CI: 2.01–2.94) for OS with no significant heterogeneity (χ^2^ = 14.39, *p* = 0.639; I^2^ = 0%), indicating the robustness of our results.

### Publication bias

**Fig. 5:**
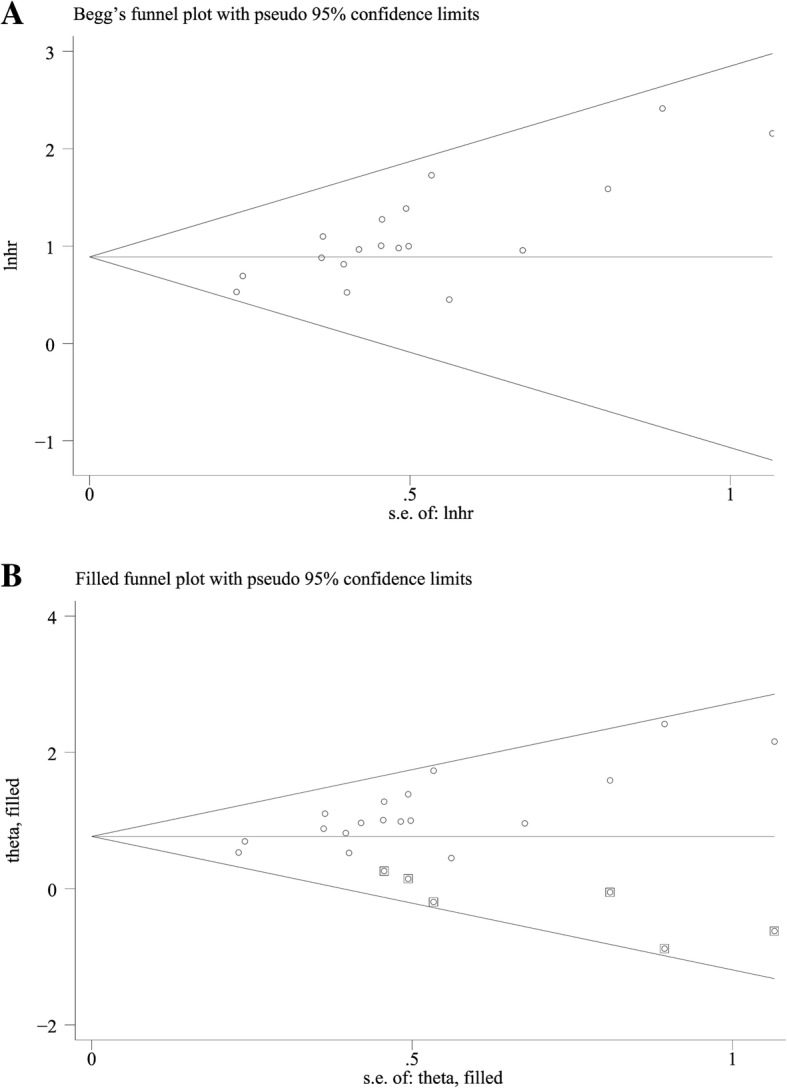
Publication bias of the enrolled studies. **a** Funnel plot, **b** Trim and fill method

Begg’s test was performed to estimate the publication bias of the studies in this meta-analysis. The study of Gabitass, R.F et al., 2011 was excluded based on the results of sensitivity analysis. The asymmetric funnel plot (Fig. 5a) and the result of Begg’s test (*p* < 0.0001) indicated the possibility of publication bias. Thus, the trim and fill method was performed, and the pooled HR of 2.19 (95%CI: 1.78–2.69) remained statistically significant (Fig. 5b).

## Discussion

Numerous studies have stressed the biological importance of tumor microenvironment in carcinogenesis and progression. Previous studies have lay solid foundation on the association between increased MDSCs level and poor prognosis in various types of tumor, including melanoma [8, 9], GI cancers [10–12], NK/T lymphoma [13], bladder cancer [14], small cell lung cancer [20], etc. However, results of these studies are not comparable, owing to the different design, patient population, and therapeutic strtegies, and the diversity in cut-off value defining “elevated MDSCs”. Our meta-analysis presented here was the first study assessing the association between elevated MDSCs level and prognosis in cancers, including solid tumors, as well as hematological malignancies. Sixteen trials with a total of 1864 patients were included in this meta-analysis. The combined data indicated that elevated MDSCs level was significantly associated with shorter OS, and poor DFS/RFS of patients with various cancers.

Obvious heterogeneity existed among the included studies in our meta-analysis (I^2^ = 61.3%, *P* < 0.01). Subgroup analyses were conducted to find out the source of heterogeneity. The heterogeneity for OS decreased in the subgroup analysis based on cancer types. It implied that cancer types might contribute to heterogeneity to some extent. Moreover, the sensitivity analysis identified the study of Gabitass, R.F et al., 2011 affecting the results obviously. And the heterogeneity decreased after excluding the outlier study.

The complex association between chronic inflammation and tumor development has commenced to be investigated during the last decade [21]. Chronic inflammation is considered to mediate tumor progression via immunosuppression, releasing various cytokines and recruiting several immunosuppressive cells, particularly MDSCs [2, 22]. Recently, several studies have shown that MDSCs are associated with poor progression in solid tumor and hematologic malignancies [8, 11, 23–25]. Mechanically, MDSCs expansion inhibits T cell proliferation, decreasing cytokine secretion, recruiting regulatory T cells, as well as prohibiting natural killer cells (NK cells) activation, thus hampering the host anti-tumor immune response [5, 26, 27]. In addition, MDSCs also exert non-immunological functions by promoting angiogenesis, accelerating tumor invasion, and metastasis [28, 29]. Recently, some studies have explored the direct interaction between MDSCs and tumor cells [7]. Therefore, increased MDSCs frequency might generate a favorable immune microenvironment, contributing to poor prognosis in cancer patients.

It is notable that this meta-analysis has some limitations; therefore cautions are called for interpreting the results. First, only 16 studies were included in this meta-analysis. Besides, the result of Begg’s test indicated the possibility of publication bias. Second, the diversity of cut-off values defining high MDSCs frequency in each study might contribute to heterogeneity among the enrolled studies. Third, some studies did not directly report HR or 95%CI, possibly leading to inaccurate estimation of HR and 95%CI. Forth, differences in paper quality and study design might cause bias to some extent. Fifth, our results might overestimate the prognostic role of MDSCs with positive results from most of the included studies.

## Conclusion

Here, several electronic databases, including Pubmed, Embase and Web of Science, were searched for related studies, and 16 studies with 1864 patients were enrolled in the first meta-analysis estimating the association between elevated MDSCs level and survival outcomes of patients with various types of cancers. Taken together, we can draw a conclusion that MDSCs gains a prognostic value for cancer patients. More multi-center prospective cohorts and longer follow-up period are warranted to further validate the prognostic role of the MDSCs in cancer patients.

## Abbreviations

CI: Confidence interval
DFS: Disease-free survival
EFS: Event-free survival
GI cancers: Gastrointestinal cancers
HR: Hazards ratios
MDSCs: Myeloid-derived suppressor cells
NOS: Newcastle–Ottawa Quality Assessment Scale
OS: Over survival
RFS: Recurrence-free survival
